# Trauma to Transformation: the lived experience of bereaved parents of children with chronic life-threatening illnesses in Singapore

**DOI:** 10.1186/s12904-020-00555-8

**Published:** 2020-04-06

**Authors:** Oindrila Dutta, Geraldine Tan-Ho, Ping Ying Choo, Xinyi Casuarine Low, Poh Heng Chong, Carolyn Ng, Sashikumar Ganapathy, Andy Hau Yan Ho

**Affiliations:** 1 Psychology Programme, School of Social Sciences, Nanyang Technological University, Singapore, Singapore; 2 HCA Hospice Care, Singapore, Singapore; 3 Children’s Cancer Foundation, Singapore, Singapore; 4 Portland Institute for Loss and Transition, Portland, Oregon, USA; 5 Club Rainbow Singapore, Singapore, Singapore; 6 Centre for Population Health Sciences, Lee Kong Chian School of Medicine, Nanyang Technological University, Singapore, Singapore; 7 Palliative Care Centre for Excellence in Research and Education, Singapore, Singapore

**Keywords:** Bereavement, Pediatric palliative care, Rituals, Qualitative research, Asia

## Abstract

**Background:**

In 2016, over 6.6 million children died globally, and 245 children died in Singapore. Chronic illnesses are prevalent causes of child mortality around the world. Despite growing research that examines the lived experience of parents bereaved by their child’s chronic life-threatening illness, there is no such study within the Asian context.

**Methods:**

To bridge this knowledge gap, meaning-oriented, strength-focused interviews were conducted with 25 parental units (i.e. 6 couples, 13 lone mothers, 4 lone fathers, and 2 primary parental figures) who lost their child to chronic life-threatening illness in Singapore (*N* = 31), including those of Chinese (*n* = 17), Malay (*n* = 10) and Indian ethnicities (*n* = 4), between August 2017 and April 2018.

**Results:**

Data analysis adhering to the grounded theory approach revealed 7 themes and 25 sub-themes that were organized into a Trauma-to-Transformation Model of Parental Bereavement. This model shows the major milestones in participants’ lived experience of their child’s chronic life-threatening illness and death, starting from the diagnosis of their child’s chronic life-threatening illness and the subsequent emotional turmoil (Theme 1), the mourning of their child’s death and the losses which accompanied the death (Theme 3) and participants’ experience of posttraumatic growth through reflection of their journey of caregiving and child loss (Theme 5). The model further describes the deliberate behaviors or ‘rituals’ that helped participants to regain power over their lives (Theme 2), sustain an intimate bond with their child beyond death (Theme 4), and transcend their loss by deriving positive outcomes from their experience (Theme 6). Finally, the model denotes that the lived experiences and well-being of participants were embedded within the health-and-social-care ecosystem, and in turn impacted by it (Theme 7).

**Conclusion:**

These themes and their corresponding sub-themes are discussed, with recommendations for enhancing culturally sensitive support services for grieving Asian parents around the globe.

## Background

In 2016, over 6.6 million children died globally [1]. Chronic illnesses are a prevalent cause of child mortality [2]. Although childhood deaths in Singapore (age < 19) decreased over the years to 245 reported in 2016 [3], it is worrying that deaths due to chronic conditions climbed from 120 in 2014 to 152 in 2016 [4]. A child’s death shatters parents’ assumption that children will never die before their parents, and when they do, it invalidates parental status and roles [5]. Moreover, child loss is associated with disenfranchised grief, that is, the magnitude of loss that mourning parents experience is not recognized by society [6]. Bereaved parents are also at greater risk of physical, psychological and social health problems, especially in the initial months after child loss [7, 8].

Caring for a child with a chronic life-threatening illness is described in literature as ‘battling the dragon’ [9]. This is because the stress brought on by the cycle of treatments and sometimes relapse is multifaceted, including practical and financial demands of caregiving [10], strain in parents’ marital relationship [11], and neglect of other healthy children [12, 13]. Further, parents caring for their child with a chronic life-threatening illness must communicate often and liaise closely with medical professionals, but such interactions could increase parents’ level of distress if they find themselves feeling alienated from decisions surrounding their child’s care and treatment [13, 14].

The meaning reconstruction framework proposed that ascribing an explanation to the loss (sense-making) and discovering personal or social gains as an outcome of the loss (benefit-finding) is associated with healthy adaptation [15]. It is also known that processing emotions and constructing new meanings surrounding loss can be helpful for healthy individuals who display enduring grief reactions subsequent to loss [16]. Moreover, highly challenging life-events such as the loss of a loved one can bring about positive change in individuals, termed as posttraumatic growth [17, 18]. There is evidence for meaning reconstruction and posttraumatic growth among bereaved parents of chronically ill children [19–21]. However, because of limitations in their scope and sample, it is unknown whether findings from these studies are universally applicable.

Other research has examined ritualization in grieving processes. Rituals (defined as repetitive or one-time behavior that give symbolic expression to certain feelings and thoughts of the individual) possess healing properties, including the ability to provide expression to strong emotions and a sense of structure [22]. When faced with a loved one’s death, rituals can help survivors cope with the physical and psychological demands of loss and offer meaning and legitimacy to life transitions [23]. For bereaved parents, ritualization following child death can help to mediate grief through maintaining continuing bonds with their deceased child, providing a sense of control and offering a means of posttraumatic growth by honoring and memorializing their child [24]. Dutta et al. explored the lived experience of parental bereavement due to a child’s chronic life-threatening illness and subsequent death in a qualitative systematic review of 25 high-quality articles published between 2000 and 2017, and highlighted that there were no Asian studies conducted on the parental bereavement experience stemming from a child’s death due to chronic illness [25]. This means that the unique needs and concerns of Asian bereaved parents remains unknown, creating a knowledge gap in the delivery of to Asian parents.

### Present study and research questions

The present study aims to bridge the knowledge gap identified by Dutta et al. by comprehensively understanding the lived experience of bereaved parents in Singapore from the time of their child’s diagnosis through bereavement, thereby serving as a first-of-its kind Asian study to inform the development of culturally-sensitive holistic support services for parents facing impending and actual child loss. The overarching research questions were: (1) How do parents in Singapore journey from the diagnosis of their child’s chronic life-threatening illness to his/her death? (2) How do bereaved parents in Singapore experience life after their child’s death? (3) How can psychosocial care be enhanced so that bereaved parents of children with chronic life-threatening illness in Singapore better cope with their experience of loss, and be provided with opportunities for meaning making leading to posttraumatic growth? In this study, the term ‘chronic’ refers to an illness that is prolonged and rarely cured [26], and the term ‘life-threatening’ describes illnesses characterized by prognostic uncertainty and little consensus among experts regarding which conditions have a reasonable hope for cure [27].

## Methods

### Research approach and design

A constructivist paradigm was adopted to elicit narratives of grief and bereavement among parents who suffered the loss of their child due to a chronic life-threatening illness and explore the meaning they ascribed to their lived experiences of grief and loss. The objective was to examine patterns and ascertain commonalities within the participants’ narratives [28]. A phenomenological stance was adopted, which honored participants’ experiences and their ability to find and create meaning through them, and facilitated dialogue between the participant and the researcher. Such a co-constructive process generated findings with ‘moderatum generalization’, which implies that the findings can be replicated in a situation with comparable physical features and shared cultural norms and values as those of participants in this study [29].

### Sampling

A method of purposive sampling was employed. In liaison with three community collaborators, namely HCA Hospice Care (HCA), Children’s Cancer Foundation (CCF), and Club Rainbow Singapore (CRS), family units of lone or couple parents who lost their child to a chronic life-threatening illness were invited to participate in the study. This sample frame enabled the researchers to examine the experience of parental bereavement due to a range of chronic life-threatening illnesses. A parent was defined as the child’s primary family caregiver with whom the child fostered a close bond. The target sample size was 25, which is deemed appropriate by previous studies exploring lived experience and the concept of data saturation [30]. The inclusion criteria for parents to be considered for the study were: (1) previously had a child diagnosed with a chronic life-threatening illness between the ages of 0–19 years, (2) their child had passed away due to such conditions in the subsequent years, and (3) a minimum period of 6 months had elapsed between their child’s death and the study interview. Parents who were not able to communicate in English, Mandarin or Malay, were not able to provide informed consent, or showed signs of depressive symptoms or any other major mental illness were excluded from the study.

### Participant recruitment

Potential participants were identified and contacted by the appointed staff of HCA, CCF and CRS for a telephone screening to explain the study. This telephone screening ensured that participants fit the sample frame and had the capacity to engage in the study. Those who agreed were referred to the research team, who then contacted the participants via telephone to arrange an interview appointment. The research team received 31 referrals, of which 27 families agreed to participate. Two family units showed depressive symptoms during the interview; their data were excluded from the final analysis and concerns about their well-being were shared with the referral organization for subsequent follow-up. No demographic differences were noted between the families that refused to participate and the families that were deemed ineligible as compared to the families that were studied as part of this investigation.

### Data collection

Inspired by the meaning reconstruction interview framework [31], this study provided a platform for participants to verbalize their bereavement experiences. A semi-structured interview schedule was developed covering domains on participants’ experiences of caring for their child with a chronic life-threatening illness, the evolution of their relationships throughout this process, coping with end-of-life, coming to terms with their child’s death, their experiences with health-and-social care systems, adjustment to loss and grief, and the types of support that helped them to cope (see Table 1). The researcher enquired about their subjective appraisal of those events, and the underlying beliefs or values that affect their appraisals. Such meaning-oriented interviews were first pilot tested and subsequently carried out with one family unit at a time, with either one or both parents present, by a pair of researchers from the research team. Participants completed an informed consent form before engaging in a 60 to 90-min interview; upon completion of each interview, a debriefing session was held between the researchers to review their experience and impressions of the shared narratives. The interviews were conducted between August 2017 and April 2018. The venue of the interviews was either a private interviewing room at HCA or at the participants’ respective homes. Each interview was audio-recorded and transcribed verbatim. Ongoing reviews of transcripts were carried out by the first author for quality assurance. Twenty-two interviews were conducted in English; three interviews were conducted in Mandarin and their transcripts were translated to English and verified by two researchers for accuracy. All interviewed participants received a S$30 cash voucher each as an appreciation for their time.

**Table 1:** Meaning-oriented semi-structured interview guide

1. Could you please describe to me what your life looked like prior to [child’s name] diagnosis?	
2. What has life been like for you after [child’s name] was diagnosed with the illness?	
3. What was your experience like providing care to [child’s name]?	
4. How has your relationship with [child’s name] changed throughout the process of illness?	
5. What was your experience like as [child’s name] reaches the final phases of life?	
6. How has your life changed after the passing of [child’s name]?	
7. What was your experience like with the health and social care system throughout this journey?	
8. Looking back at this experience, what helped you along your journey of caregiving and bereavement?	
9. If you have the opportunity to speak with someone who is currently going through a similar situation as you had, what kind of advice would you give to them?	
10. As a parent who has been through this experience, is there a message that you would like to share with the world?	

### Data analysis

**Fig. 1:**
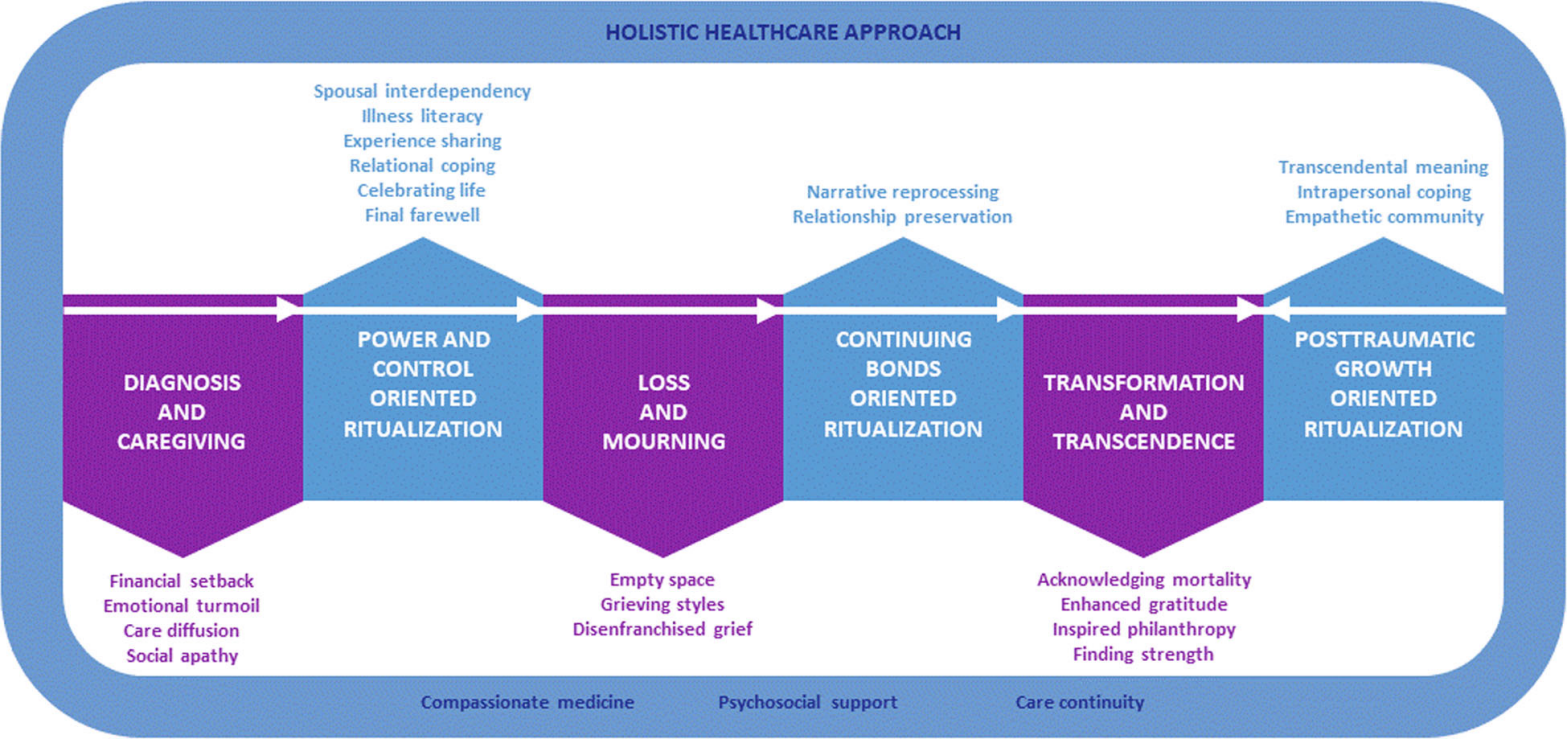
Trauma to Transformation: The lived experience of Asian bereaved parents of children with chronic life-threatening illness

All audio recordings were transcribed using Microsoft Word and these transcriptions were in turn imported into QRS NViVo 11. Data analysis adhered to the grounded theory approach [32] which involved line-by-line coding, axial coding and selective coding to generate conceptual themes. In the final stage of analysis, all major categories, themes and sub-themes were operationalized. Relationships between themes and sub-themes were proposed and mapped with supporting quotes from transcripts to generate a model (see Fig. 1). To ensure research rigor and trustworthiness of findings, stringent mechanisms were adopted including maintenance of an audit trail, peer debriefing, checking preliminary themes and interpretations against data obtained in subsequent interviews, inter-researcher consensus in finalizing of themes, achievement of data saturation and theory triangulation [33, 34].

### Ethical considerations

This study met the ethical approval criteria of Nanyang Technological University’s Institutional Review Board [IRB-2017-03-044]. The principal investigator and research team were qualified to provide on-site emotional support for research participants during data collection. A referral system for emotional support was developed with HCA, CCF and CRS so that participants could obtain further psychosocial support if required.

## Findings

The sample comprised 6 couples, 13 lone mothers, 4 lone fathers and 2 primary parental Figs. (*N* = 25 parental units). The mean age of the participants was 49.35 (SD = 10.47). All participants were Singapore citizens or Permanent Residents. Table 2 provides an overview of the participants’ sociodemographic details.

**Table 2:** Demographic information of participants

	Age (in years)	Sex	Marital status	Ethnicity	Child’s diagnosis	Child’s age at diagnosis (in years)	Child’s age at death (in years)	Time since child’s demise (in years)	Number of surviving children
P1	50–59/50–59	M/F	Mar/Mar	Ch/Ch	Cancer	10–14	10–14	2	1
P2	50–59/50–59	M/F	Mar/Mar	Ch/Ch	Cancer	15–19	15–19	2	2
P3	50–59	F	Mar	Ch	Cancer	0–4	25–29	1	2
P4	60–69/50–59	M/F	Mar/Mar	Ch/Ch	Cancer	10–14	15–19	2	1
P5	40–49	F	Mar	Ma	Cancer	Undisclosed	15–19	4	3
P6	40–49/30–39	M/F	Mar/Mar	Ma/Ma	Cancer	0–4	5–9	2	2
P7	30–39	F	Mar	In	Cancer	0–4	0–4	1	2
P8	30–39	F	Mar	Ma	Cancer	10–14	10–14	2	2
P9	60–69	F	Sin	In	Muscular dystrophy	5–9	15–19	0.6	1
P10	40–49	F	Mar	Ch	Cancer	10–14	10–14	4	2
P11	40–49	M	Wid	Ch	Undisclosed	0–4	10–14	1	1
P12	40–49	M	Mar	In	Brain tumor	10–14	10–14	4	1
P13	40–49	F	Mar	Ch	Brain tumor	0–4	0–4	4	1
P14	40–49	F	Div	Ma	Cancer	0–4	5–9	4	3
P15	40–49	F	Mar	Ma	Cancer	10–14	10–14	5	3
P16	60–69/60–69	M/F	Mar/Mar	Ma/Ma	Kidney failure	0–4	15–19	3	1
P17	50–59	F	Mar	Ch	Cerebral palsy	0–4	15–19	2	2
P18	50–59	F	Mar	Ch	Blood clot in the brain	5–9	15–19	3	–
P19	30–39/30–39	M/F	Mar/Mar	Ch/Ch	Spinal muscular atrophy	0–4	0–4	2	–
P20	40–49	F	Mar	In	Cerebral palsy	0–4	15–19	1	2
P21	70–79	M	Wid	Ch	Multiple diagnoses	0–4	30–34	1	1
P22	30–39	F	Mar	Ma	Multiple diagnoses	0–4	0–4	0.5	2
P23	50–59	F	Mar	Ma	Cancer	0–4	5–9	0.6	–
P24	40–49	F	Mar	Ch	Congenital heart disease	0–4	10–14	3	–
P25	30–39	M	Mar	Ch	Cancer	0–4	0–4	0.6	1
Mean	49.35					4.73 y	12.06 y	2.21 y	1.44
SD	10.47					5.05 y	7.21 y	1.36 y	0.92 y

Note. *M* Male, *F* Female, *Mar* Married, *Sin* Single, *Wid* Widowed, *Div* Divorced, *Ch* Chinese, *Ma* Malay, *In* Indian, *y* years, *m* months

Figure 1 shows the major milestones in participants’ lived experience of their child’s chronic life-threatening illness and death, starting from the diagnosis of their child’s chronic life-threatening illness and the subsequent emotional turmoil (Theme 1), the mourning of their child’s death and the losses which accompanied the death (Theme 3) and participants’ experience of posttraumatic growth through reflection of their journey of caregiving and child loss (Theme 5). Figure 1 further describes the deliberate behaviors or ‘rituals’ that helped participants to regain power over their lives (Theme 2), sustain an intimate bond with their child beyond death (Theme 4), and transcend their loss by deriving positive outcomes from their experience (Theme 6). Finally, Fig. 1 denotes that the lived experiences and well-being of participants were embedded within the health-and-social-care ecosystem, and in turn impacted by it (Theme 7). In sum, Themes 1 and 2 describe how Asian parents journey from the diagnosis of their child’s chronic life-threatening illness to the end-of-life. Themes 3 and 4 explore Asian parents’ lived experience and coping in the immediate aftermath of their child’s death. Themes 5 and 6 examine Asian bereaved parents’ lived experience after the loss of their child. Finally, Theme 7 offers insight to healthcare professionals about improvements in psychosocial care and holistic health services that would positively impact Asian parents’ well-being during their child’s illness trajectory through bereavement.

### Theme 1: Diagnosis and caregiving

This refers to the reactions that surfaced in participants when they received news of their child’s chronic life-threatening illness and the struggles that they encountered through the illness trajectory and end-of-life.

#### Financial setback (number of interviews theme appeared in: *N* = 19)

Survival needs such as financial worries emerged as the most critical concern for participants after their child was diagnosed with a chronic life-threatening illness, as seen from the *N.* Participants drew attention to the high cost of treatments.

“One pill for cancer did not come cheap; one hundred dollars a day, and that added up to three thousand dollars a month.” (P11, father)

Caregiving duties often required participants to quit employment, further adding to their financial challenges.

“Ever since my son had fallen sick, I had to stop working [ … ] then my husband’s income wasn’t stable, so we started having financial issues.” (P13, mother)

#### Emotional turmoil (*N* = 16)

Participants described the dismay and disbelief which accompanied the initial diagnosis, and their worries about the long-term implications of their child’s condition.

“Shocked [...] we really cannot believe he was that sick.” (P23, primary parental figure)

“I want to know, two years later what is going to happen to her, five years later what is going to happen to her. Can she blend into society, would she be able to go to school?” (P17, mother)

Other participants expressed feeling in limbo because the process of diagnosis could be complex and prolonged.

“Me going through this for the first time, I was totally lost. And at that time, they [doctors] are not able to share more things with me. So, I feel like [ … ] don’t know who to look for.” (P22, mother)

#### Care diffusion (*N* = 14)

Participants discussed that caring for a child with chronic life-threatening illness could be an all-consuming task which resulted in them neglecting the needs of their healthy siblings. Some depended on the support of family and friends to look after healthy siblings.

“I neglected my son a little bit as I let go a lot to other people to handle him and I focused more on my daughter.” (P10, mother)

Participants also spoke of the challenges that their healthy siblings faced and regretted not having the time to attend to them.

“We went out together. I was holding her [sick child] hand, then my youngest daughter told me, ‘Mummy why every time you only hold JieJie (elder sister), you never hold me [ … ]’ Then I explained to her [ … ] She does understand, but understanding is one part [ … ] Feeling is another thing.” (P3, mother)

#### Social apathy (*N* = 11)

Participants described the societal indifference and ignorance towards their challenges. Many sick children were at the receiving end of derogatory remarks regarding their illness-related appearances.

“The children, so-called, laughed at her [ … ] said in Chinese words ‘botak’, [which] means bald.” (P3, mother)

Family members of sick children also endured insensitive social attitudes.

“The teacher [of the healthy sibling] was like ‘stop with all your stupid family story [...] don’t ask for any sympathy or be attention seeker.’” (P14, mother)

### Theme 2: Power and control oriented ritualization

As participants began to accept their child’s illness and integrated treatments and hospital visits into their lives, they adopted rituals to bring order into their seemingly uncontrollable lives.

#### Spousal interdependency (*N* = 18)

Participants developed a ritual of alternating caregiving responsibilities with their spouse, so that the child’s primary caregiver could take time off for self-care.

“When he (spouse) was not working, or when he had a few hours off, he would look after him (sick child), then I would go out for a while. Even if I only get to walk around in the lobby [of the hospital], I was happy [ … ] I had to leave for that period, for myself to take a breather.” (P13, mother)

Participants reflected that spousal support was typically in the form of pragmatic assistance and solution-focused conversations rather than in-depth discussions about inner thoughts and feelings.

“Talk, not so much. [We were] physically present [for each other] as much as possible. Functional needs first, survival mode. [We] talked a bit about the diagnosis, or what we thought was the best place to go or the next step to go on.” (P1, father)

#### Illness literacy (*N* = 15)

Participants employed varied resources to obtain more information about their child’s condition.

“We went there (Singapore Cancer Center). I did my homework. I asked the doctor dozens of questions.” (P2, mother)

They felt that learning about the illness would help them to collaborate with medical professionals and make informed decisions that were most appropriate for their child.

“You research some stuff, you try to bring up to the doctor [...] like for example, we brought up the use of the Cough Assist and eventually they allowed us to use it.” (P19, mother)

#### Experience sharing (*N* = 13)

Participants often turned to parents of other children suffering from similar health conditions to seek support and exchange knowledge.

“We talk to them (parents of other sick children) [about] what are we expecting, the daily needs that we need to do for them [ … ] if our equipment breaks down, what do we do?” (P19, mother)

Parents who were more experienced played the role of comfort-providers for “newcomers” who were experiencing heightened levels of anxiety and uncertainty.

“We extended our help or talked to those ‘newcomers’ warded in the hospital. We spoke to them about our experience, such as the procedures. Most of them didn’t know what to do [ … ] so we shared our experiences with them.” (P4, father)

#### Relational coping (*N* = 10)

Participants faced their child’s chronic life-threatening illness together with their family, which enhanced their perceived power in coping with illness-related challenges. Often, this translated into harnessing strengths of different family members so that each person could feel involved in caring for the sick child.

“I want my parents to feel that they are involved in making him recover by cooking for him [ … ] because we [husband and wife] don’t have time to cook.” (P2, father)

By spending quality time together as a family, participants coped with the challenges of their child’s illness such as prolonged hospital admission.

“Daddy makes sure that at any point where they have free time, he will grab the opportunity and bring everybody together to the hospital. Although it’s not a happy place, it’s where the family would be together.” (P6, mother)

Participants also conveyed their emotional and practical availability to other family members who found it difficult to care for the sick child.

“My daughter needed help at that time, she was alone [ … ] She has to work and then who’s going to look after my grandson? So, I felt it was my duty to look after him.” (P23, primary parental figure)

As their child’s illness worsened and hopes for recovery were replaced by preparation for mortality, participants continued to engage in rituals which helped them to feel a greater sense of control over their lives. The next two sub-themes reveal the ways in which participants asserted their power in the final stage of their child’s life.

#### Celebrating life (*N* = 18)

Participants endeavored to help their child celebrate life, transcend illness-related challenges and fulfill their child’s desires.

“We went to shop for baking stuff, after which we booked a café. Then there was a so-called chef and all the waiters came down, taught him cake-making. There was a party, everyone joined and made ice cream.” (P25, father)

They thus experienced the satisfaction of fulfilling their parental role by spending quality time with their child and celebrating life in his/her final days.

“Try to fulfill whatever wish they wish for [ … ] once they are gone, you are not able to do all these things.” (P5, mother)

#### Final farewell (*N* = 22)

Participants continued to fulfil their parental role in the final moments of their child’s life through comforting acts and alleviating his/her fears about dying.

“When I hold on to him, when daddy holds on to him, we spoke to him and we told him that it was going to be alright.” (P7, mother)

For children whose death was predicted from the gradual decline in their body functions, participants planned for their child to be brought home from the hospital and for loved ones to visit and bid goodbye.

“I called everyone [ … ] they came to say their final goodbyes to him [ … ] Then Saturday, he left us at 3 am.” (P13, mother)

Participants whose child had an unexpected death were regretful for not having the time and space for a farewell.

“I feel very guilty [...] I’m thinking that I’m not beside him at that time, maybe we don’t know what is in his mind. Maybe the few days before he passed away, I can take leave and stay with him [ … ] I didn’t know he would leave me very soon, I thought it was going to be like every day.” (P20, mother)

### Theme 3: Loss and mourning

In the aftermath of their child’s death, participants gradually began to process the complex amalgamation of emotions that accompanied the loss.

#### Empty space (*N* = 18)

Participants described the loss of their child as the creation of a void that could never be filled.

“There’s always that empty space here. Even after he was buried, sometimes my husband says, ‘Eh we used to call them all three (children) to come for dinner right, now we can only call two.’” (P8, mother)

This void was particularly pronounced for the parent who was the child’s full-time primary caregiver.

“Like every other day you’ll be planning your schedule because he has a medical appointment [...] and now suddenly you no longer have to plan for him.” (P7, mother)

Bereavement brought along the realization that participants had lost not only their child but the entire world they had constructed around caregiving.

“The house became very quiet. When she was around with the equipment all that, at least you can hear the sounds of the machines beeping [ … ] so after that suddenly no machine sounded, no nothing.” (P19, mother)

#### Grieving styles (*N* = 10)

Parents in a spousal relationship noted that they had different ways of mourning their loss.

“He is quieter about it and I want to talk about it. But then when I want to talk to him about it, he doesn’t, he’s not ready.” (P6, mother)

Participants reflected that such variation could reflect personality factors and the nature of the parent’s involvement in raising the child rather than gender.

“Maybe it’s not just about men and women. It’s about character … My husband likes to talk. I don’t like to talk much. I write [...] but if I were a stay-at-home mother, that bond would be very close. But I don’t. So that feeling is not the same as him. He has many beautiful memories rather than me. He feels the loss more than me.” (P15, mother)

#### Disenfranchised grief (*N* = 8)

The intensity of bereaved parents’ anguish and the significance of their loss often did not receive societal validation.

“They (refers to her husband’s aunts) told me, ‘You should try for another child with your husband.’ I don’t want to.” (P18, mother)

Some participants recalled that outward expressions of their intense loss were deemed inappropriate.

“Every day I sit alone, I cry [ … ] my son notices. He says, ‘Please, what are you looking and crying for? Over already now.’” (P9, primary parental figure)

### Theme 4: Continuing bonds oriented ritualization

Maintaining psychological and spiritual proximity with their child helped participants cope with the sense of helplessness created by his/her death.

#### Narrative reprocessing (*N* = 20)

Participants ascribed a sense of personhood, meaning and purpose to their child’s life through an evolving narrative of his/her life story.

“He got to spend time with his family, he got to celebrate his father’s birthday. He got his friends to come and visit. He got to make sushi with one of his friends who had the same diagnosis as him.” (P8, mother)

Many participants spoke with pride about the strength and fortitude that their child displayed despite the pain and suffering that he/she endured.

“He was pretty strong even though how bad the chemo went, the side effects came in, vomiting all this, he is accepting it quite well.” (P2, father)

Remembering their child’s inspirational attributes thus helped participants search for personal growth in their loss.

“And through these 15 years, along the way, when I’m down and out, I look at her. Why is it she (deceased child) can enjoy life even in that state? Why am I so upset over small things or things that I can control, or I can get rid of?” (P17, mother)

#### Relationship preservation (*N* = 12)

Participants continued to acknowledge and preserve their relationship with their deceased child regardless of his/her physical presence.

“When people ask me how many children I have, I will reply saying, ‘I have two children.’ ‘Where’s the older child?’ ‘He passed away.’ I don’t want to say that I only have one son now. I want to acknowledge him.” (P13, mother)

Participants also shared their wish to memorialize their child in a way that the world would learn about his/her positive attributes.

“I want people to read his story, his goodness, whatever he went through, the positive things that we achieved [ … ] I want to put all that into one book.” (P15, mother)

### Theme 5: Transformation and transcendence

Participants described a sense of transformation that they were able to achieve as they reflected on the traumatic nature of the journey they had been through, including their experience of caregiving throughout their child’s illness trajectory, end-of-life, the loss of their child and finally their sense of transcending this trauma.

#### Acknowledging mortality (*N* = 11)

Participants reported that the experience of child loss had triggered reflection about the fragility of human life in a world that is unpredictable and uncontrollable.

“Life can be too short. God can just take [loved ones away] anytime.” (P6, father)

This often resulted in re-evaluation of their lives and greater appreciation of aspects they had previously taken for granted, particularly their personal relationships.

“I’m very career driven in the past. Now family is more important... the time I spend with my daughter, the time I spend with my wife.” (P25, father)

#### Enhanced gratitude (*N* = 11)

Participants spoke of their increased ability to observe and appreciate the positive aspects of their lives.

“We’re very lucky to be here in Singapore, we got so much help from people, all walks of life, from different kinds of races also.” (P23, primary parental figure)

This gratitude in turn enhanced their sense of well-being, reduced feelings of regret and facilitated acceptance of the loss.

“It is not a sudden death. It’s a period where you can have that bonding, that moment, that quality time, instead of just being abruptly taken [ … ] God gave you that period you have to be with her, enjoy every second so you don’t have regrets.” (P14, mother)

#### Inspired philanthropy (*N* = 9)

Participants found that serving others helped them to find meaning in their loss.

“She [wife] does a bit of administration for them [welfare organization] [ … ] Some of our weekends are spent going to some of the beneficiaries’ families [ … ] we found great meaning in it so yeah, we help out in this cause.” (P19, father)

They explained that the challenges they had encountered during the caregiving period had made them more sensitive to the needs of others, which now drove them to help others whenever an opportunity presented itself.

“We were also moving with wheelchairs and we had difficulties. So now I see old people moving on their own, I go and help automatically.” (P12, father)

Others described feeling indebted to the organizations from whom they had received support, and now felt it was their duty to help in whatever way they could.

“Right now, I volunteer at the center two times a week [ … ] My grandson benefited a lot from the session in school, received a lot of love from the staff and teachers from the center, so I feel I have to give what I can.” (P23, primary parental figure)

#### Finding strength (*N* = 8)

Many participants reported greater strengths and resilience as an outcome of their challenging journey of caregiving and loss.

“I realized that I have become more cheerful [ … ] A lot of things are actually unimportant, and I can choose not to care about them [ … ] I feel that my worldview has gotten bigger too.” (P13, mother)

The ability to persevere through the challenges they had encountered during caregiving for their sick child brought about the realization of the immense strength they had within themselves.

“After all this, I’m really a warrior mother. I'm not like others, I'm more than others so I can do much better than others.” (P20, mother)

### Theme 6: Posttraumatic growth oriented ritualization

Although the intense anguish of child loss tended to reduce over time, the memory of their child and the sadness of losing him/her was relatively permanent. This enduring pain served as a catalyst for participants to continue to engage in rituals which empowered them to bring about positive change and growth outcomes in their lives. Such rituals further enhanced participants’ experience of transformation and transcendence in their journey of grief and loss, as represented by the bidirectional arrow in Fig. 1.

#### Transcendental meaning (*N* = 23)

Participants’ continued to transcend their grief each day by focusing on the bigger picture beyond their child’s death. Such transcendental meanings were sometimes person-centric, wherein participants’ personal values and beliefs shaped their understanding of their child’s death.

“Even though today he passed away, tomorrow the sun still rises. So, all of you got to accept the fact that he is not going to be here. Things carry on.” (P2, father)

For other participants, faith-based beliefs contributed to their perception of child loss.

“The greatest comfort I have is that I know she is with Jesus.” (P3, mother)

Still others explained their child’s death as an end to his/her pain and suffering.

“If you cannot make him better, you take him away [ … ] you take him away rather than I see him suffer.” (P15, mother)

#### Intrapersonal coping (*N* = 14)

Participants discussed their personally relevant strategies to cope with their grief as well as express and process their emotions.

“It’s only when I drive, I go back from [location name], it’s a long way right? 1 hour, 35 minutes sometimes. That is where (makes crying sound).” (P15, mother)

“The nearest park here is [name of Park]. So, there is a favorite spot for me that I will go to. I’ll spend time reading the Bible, I’ll bring a book and start to write [ … ] as I write, I cry.” (P1, mother)

Others explained that they coped with their grief by occupying their time with activities.

“Through work, I gradually learnt not to think about him. At home, I occupy myself with housework or I go out and walk around [ … ] this is how I get by day by day.” (P18, mother)

#### Empathetic community (*N* = 13)

Participants found it helpful to actively participate in support groups that brought them closer to other parents with similar grief experience.

“We have a closed group of us grieving parents. I gained help from there. They are all parents who lost their children in any way, in any form, at any age. But all feel the same pain.” (P3, mother)

They explained that the distinctive nature of a bereaved parent’s emotional pain could only be validated by somebody who had experienced it firsthand.

“Only those who have gone through this would understand.” (P18, mother)

Participants who had come to terms with their loss found meaning by joining such groups, as a means of demonstrating empathy and encouragement for others.

“I don’t mind talking to them or motivating them in life and telling them about things that I went through.” (P14, mother)

### Theme 7: Holistic healthcare approach

The relationship between participants and their healthcare providers, and participants’ perception of their healthcare provider’s compassion and care towards their child affected their personal well-being.

#### Psychosocial support (*N* = 19)

Participants were appreciative of the psychological and emotional care that they received from their social workers and counsellors at the time of caring for their chronically ill child.

“I really appreciate [them]. They counselled the girl (sick daughter). They counselled me [...] be there for me to talk about whatever I want to talk.” (P15, mother)

They felt they could openly share their fears and anxieties, which was described as vital to mental well-being.

“Sometimes they (parents) also need vitamins. Vitamins are not like A, B vitamins. It’s like support [ … ] counseling, motivating.” (P20, mother)

Psychosocial support aided participants to transition from the role of a caregiver to that of a bereaved parent.

“They come and visit and see what they can help (during child’s wake) … They also asked me if I could let them know if I needed any help.” (P24, mother)

#### Compassionate medicine (*N* = 17)

Participants perceived that there was a need for physicians to show greater compassion for their patients and respect the family’s wishes for quality of life for their child.

“They saw the children as patients. They never treated them as children. Whenever they needed to poke the children to find veins and these types of things, they were rough.” (P7, mother)

“The doctor wanted to save his life, not his leg. But to us parents, I rather let him have a leg. Since it [cancer] cannot be cured I rather let him enjoy the rest of his life.” (P2, mother)

Others noted that a compassionate physician would have the capacity to show greater respect to the child and view the suffering from the child’s perspective.

“This doctor, very fresh in the morning, ‘How is your pain, [on a scale of] 1 to 10?’ My son said, ‘100’. ‘Couldn’t be a 100.’ When he said the pain is 100, you can say it’s very painful, right? How could you say it couldn’t be like that? Did you feel his pain? Have you ever had cancer and relapse before?” (P8, mother)

#### Care continuity (*N* = 14)

Participants favored an integrated approach to their child’s care that comprised disease-directed treatments together with emotional support and comfort provision.

“When I say some with passion, while checking on her, they played with her, that kind of thing, to make her feel more comfortable.” (P19, mother)

However, only a small proportion of parents perceived themselves to be recipients of such holistic care, as can be seen from the number of parents who emphasized the need for greater compassion in medical care. Additionally, participants also expressed that a quality healthcare service would involve clear, adequate and consistent communications from the physician regarding their child’s treatment and prognosis.

“Some people tend to explain it in a very technical jargon form [ … ] what do you mean by MRD, minimal residual disease? What do you mean by blood peripheral blast? These kinds of terms sound very intimidating at times.” (P25, father)

## Discussion

This is the first known Asian study to critically examine the lived experience of parents bereaved by their child’s death due to a chronic life-threatening illness. The sample comprises Singapore-based middle-aged parents whose children suffered from a wide variety of conditions ranging from cancer to congenital conditions, with the caregiving period lasting between 5 months to 31 years. The narratives obtained from this demographically diverse sample provides invaluable insight into the major milestones that formed the parental bereavement trajectory, and the rituals parents adopted to confront the challenges that life presented. The findings also reinforce the need to upgrade the health-and-social-care system within which participants’ experiences were rooted, while offering recommendations for enhancing global pediatric palliative services in general, and pediatric palliative services for Asian populations in particular. Additionally, the multicultural composition of Singapore society which is dominated by the major Asian racial and religious groups [33, 34] implies that findings from this Singapore-based study have moderatum generalization to other Asian societies as well.

Participants deliberately engaged in ‘rituals’ which empowered them to play an active role in shaping the course of their lives, both before and after their child’s death. This impact of rituals in helping mourning parents maintain continuing bonds with their deceased child, assume power and control over their lives and facilitate posttraumatic growth is supported by other parental bereavement studies [22–24]. Further, participants developed new meaning structures in response to the distress caused by their child’s death including benefit-finding in their experience, positive re-appraisal of their trauma, maintaining continuing bonds with their deceased child, cultivating a new outlook in life and moving towards personal growth. This aligns with previous research on meaning reconstruction in bereavement [15, 31, 35]. Finally, the evolution and interpretation of bereaved parents’ narratives of their child’s life story facilitated the addition of meaning to their stories of suffering, a finding which resonated with narrative identity theory [36, 37]. These experiences shared by participants point to important service considerations for pediatric palliative service providers, so that the needs of children with chronic life-threatening illness and their families can be effectively met.

Findings from this Asian study where participants were of predominantly Chinese origin, followed by Malay and Indian origin, build on our previous work in parental bereavement which focused on participants from a Western context [25]. An important contribution of the present study is its value-addition to the literature on family support for parent-caregivers and bereaved parents. The data revealed that Asian parent-caregivers who were caring for their child living with a chronic life-threatening illness often relied on their spouse for pragmatic support to cope with caregiving *(Sub-theme: Spousal interdependency)* as well as on their extended family members and friends who had a collaborative attitude towards care provision for the sick child *(Sub-theme: Relational coping)*. Thus, for the Asian parents in this study, support provided by their spouse and extended family members was an important resource in their caregiving journey and tended to be instrumental in nature (for example, acts such as stepping in to provide respite care or cooking meals). This finding corroborates other studies on how Asian families cope with a problem - through pragmatic means that focus on finding a solution rather than exploring feelings and causes [38, 39]. As yet, it is unclear how Western parent-caregivers whose children are living with a chronic life-threatening illness perceive support and assistance from their family, as the evidence seems to suggest that they may both welcome the involvement of their friends and family [9] as well as refrain from sharing their caregiving burden and obligations with people outside the immediate family [40].

On the other hand, Asian parents in this study described their disenfranchised grief following the loss of their child whereby family members did not adequately acknowledge their grief and the intensity of their suffering *(Sub-theme: Disenfranchised grief)*. In comparison, evidence suggests that emotional and psychological support from extended family members is an important coping resource for bereaved parents in the Western context [41]. It is reasonable to postulate that support from their extended family members and friends would have been helpful for Asian parents as they coped with the loss of their child, but it is possible that the uncommon nature of a child’s death and its perception as a catastrophic event [42] as well as prevalent death taboos in the Asian culture [43] prevented parents from receiving such support.

### Implications of findings

#### Working with Asian populations

The in-depth understanding of the lived experience of parental bereavement in the Asian context obtained in this study suggests that grief counselling with Asian populations must emphasize familial and social connections, which play a critical role in providing support in end-of-life care [44]. It is possible that solution-focused counseling strategies which emphasize parent-caregivers’ existing resources (such as the support of their spouse and other family members) can help to reduce the demands of caregiving and facilitate families to work together in meaningful ways. It could also be useful for clinicians to draw upon novel psycho-socio-spiritual interventions such as Family Dignity Intervention (FDI) which aim to enhance palliative care in the Asian context [45]. Although FDI has till date been studied in the context of patient-family dyads, it is possible that the FDI approach of meaning-oriented interviews can facilitate dyadic exchange for grieving parents, promote understanding of each other’s unique sense-making and coping strategies in response to child loss and establish a common ground for parents and their families to better support each other [46].

#### Enhancing psychosocial support

Findings emphasize the role of rituals in enhancing parents’ sense of control over their lives, suggesting that it would be useful for pediatric palliative care services to support parents in such rituals thereby strengthening their resilience during the period of caregiving. We recommend that this is done through provision of resources and psychoeducation for self-care, healthy family coping, harnessing available sources of support and helping their sick children live life to the fullest. Further, it is reasonable to state that a narrative approach would be best suited in this process as it would enable healthcare professionals to respectfully enquire about the effectiveness of parents’ existing resources and facilitate the mobilization of these resources to empower parents to respond to their caregiving challenges [47]. It would be important for such services to commence in early diagnosis so that parents’ memories of the days leading to their child’s end-of-life and death are filled with resilience, hope and positive relationships, in addition to improving bereavement outcomes [48]. For newly bereaved parents, findings note that psychosocial support can smoothen role transition for parents from a ‘caregiver’ to a ‘bereaved parent.’ This then implies that the pediatric palliative care team must initiate meaningful bereavement visits with the aim to provide closure to parents’ journey of caregiving, facilitate contact with support groups of parents facing similar crises and identify bereaved parents’ who are at risk for poor psychosocial outcomes [49]. Finally, the community attitudes encountered by parents in our study suggest that there is need for greater public awareness and psychoeducation on how friends and other family members can support grieving individuals. Such awareness programs could pave the path for a compassionate community that is sensitive to the unique emotional experience of child loss [41].

#### Strengthening physician-parent alliance

Findings also highlight that receiving information about their child’s illness and treatment plan in an understandable format and designing a treatment plan that aligns with the family’s goals of care for their child is important for parents facing their child’s chronic life-threatening illness. This indicates that medical staff need to engage with parents in a continuous dialogue about their child’s health-related decisions. Such dialogue must be honest, use language at a pace that parents can comprehend, must aim to collaboratively make decisions regarding goals of care for their child and take into account parents’ concerns about their child’s quality of life. By adopting these attitudes, medical staff could enhance psychological well-being for parents caring for their child with a chronic life-threatening illness [50, 51].

### Limitations and future directions

We wish to caution readers about the possibility that participants’ lived experiences described in this study were affected by retrospective bias, and it would be useful for future studies to examine the perceptions and experiences of parents as they actively cared for their child with a chronic life-threatening illness. We also acknowledge the over-representation of females in the sample and recommend that future studies focus on the lived experience of bereaved Asian fathers of children with chronic life-threatening illness. Moreover, it would be useful for future studies to maintain consistency in terms of interviewing bereaved parents either as a couple or without their spouse, an aspect that could have confounded our findings. The sample comprised parents of children with a chronic life-threatening illness diagnosed over a wide range of time, which could account for their varied experiences with their healthcare provider; future studies should consider controlling for such variability. Furthermore, the Asian parents in this study were all based in Singapore and future research is needed which examines the trajectory of parental bereavement among other Asian communities. Finally, future studies need to explore the perspectives of healthcare providers working in pediatric palliative care to form a holistic understanding of care needs, thereby offering insightful recommendations for improving service delivery. Despite these limitations, this study adds to the body of knowledge that examines how bereaved parents of children with chronic life-threatening illness cope with their grief and makes recommendations for improving pediatric palliative care delivery.

## Conclusion

Child death pervades both advanced and developing societies and impacts the physical and psychological well-being of surviving family members for the rest of their lives. Understanding what parents/families need at the final stages of their child’s life is the first step in helping them cope with bereavement. Although this study focused solely on the lived experience of bereaved parents in Singapore, there are obvious cross-cultural commonalities in the narratives of family caregivers of dying children. Acknowledgement and facilitation of rituals that are central to a family’s experience and narrative of their child’s “good death” can empower families, facilitate sustainable bonds and pave the road towards posttraumatic growth.

## Data Availability

All data collected during this study which can be made publicly available is included in this article. Additional data from the current study is not publicly available due to concerns about participants’ confidentiality.

## Abbreviations

HCA: HCA Hospice Care
CCF: Children’s Cancer Foundation
CRS: Club Rainbow Singapore
FDI: Family Dignity Intervention
